# Emerging Roles of Liquid–Liquid Phase Separation in Cancer: From Protein Aggregation to Immune-Associated Signaling

**DOI:** 10.3389/fcell.2021.631486

**Published:** 2021-06-21

**Authors:** Jiahua Lu, Junjie Qian, Zhentian Xu, Shengyong Yin, Lin Zhou, Shusen Zheng, Wu Zhang

**Affiliations:** ^1^Division of Hepatobiliary and Pancreatic Surgery, Department of Surgery, First Affiliated Hospital, School of Medicine, Zhejiang University, Hangzhou, China; ^2^NHC Key Laboratory of Combined Multi-Organ Transplantation, Hangzhou, China; ^3^Key Laboratory of the Diagnosis and Treatment of Organ Transplantation, Research Unit of Collaborative Diagnosis and Treatment For Hepatobiliary and Pancreatic Cancer, Chinese Academy of Medical Sciences, Hangzhou, China; ^4^Key Laboratory of Organ Transplantation, Research Center for Diagnosis and Treatment of Hepatobiliary Diseases, Hangzhou, China; ^5^Organ Transplantation Institute, Zhejiang University, Hangzhou, China; ^6^Shulan (Hangzhou) Hospital Affiliated to Shulan International Medical College, Zhejiang Shuren University, Hangzhou, China

**Keywords:** liquid–liquid phase separation, biomolecular condensate, cancer mechanism, protein aggregation, signaling transduction, genome stability

## Abstract

Liquid–liquid Phase Separation (LLPS) of proteins and nucleic acids has emerged as a new paradigm in the study of cellular activities. It drives the formation of liquid-like condensates containing biomolecules in the absence of membrane structures in living cells. In addition, typical membrane-less condensates such as nuclear speckles, stress granules and cell signaling clusters play important roles in various cellular activities, including regulation of transcription, cellular stress response and signal transduction. Previous studies highlighted the biophysical and biochemical principles underlying the formation of these liquid condensates. The studies also showed how these principles determine the molecular properties, LLPS behavior, and composition of liquid condensates. While the basic rules driving LLPS are continuously being uncovered, their function in cellular activities is still unclear, especially within a pathological context. Therefore, the present review summarizes the recent progress made on the existing roles of LLPS in cancer, including cancer-related signaling pathways, transcription regulation and maintenance of genome stability. Additionally, the review briefly introduces the basic rules of LLPS, and cellular signaling that potentially plays a role in cancer, including pathways relevant to immune responses and autophagy.

## Introduction

Intracellular components are compartmentalized, respectively, in living cells to facilitate the regulation of cellular activities in time and space. Apart from the traditional membrane-enclosed organelles, such as the mitochondria and endoplasmic reticulum, many organelles do not have membranous structures yet remain compartmentalized and can concentrate certain types of molecules (Handwerger and Gall, 2006; Mao et al., 2011a; Pederson, 2011). Such organelles include nuclear structures like nuclear paraspeckles, the nucleolus as well as Cajal bodies and cytoplasmic organelles such as the P-bodies, stress granules and centrosomes. Notably, membrane-less organelles such as the nucleolus, have been known since the 1830s when the structure of the cell nucleus was first described (Wagner and Barry, 1836; David, 1964). Additionally, these structures and organelles are usually composed of macromolecules such as proteins and RNA and are in many cases known as ribonucleoprotein (RNP) granules (Anderson and Kedersha, 2009). Moreover, the membrane-less compartments in the cytoplasm are involved in cellular signal transduction. For example, the Dvl protein family was initially assumed to transmit the Wnt signals in response to the binding of extracellular Wnt depending on membrane-bound vesicles. However, it was later shown that they were engaged without an enclosing membrane (Yanagawa et al., 1995; Schwarz-Romond et al., 2005; Kim W. et al., 2013).

These membrane-less organelles or structures exhibit significant liquid-like characteristics and are typically formed through a physicochemical process called liquid–liquid phase separation (LLPS) (Brangwynne et al., 2009). Given their liquid-like features, all the membrane-less intracellular organelles, structures and assemblies arising from LLPS are referred to as biomolecular condensates (Banani et al., 2017; Shin and Brangwynne, 2017). As with literal liquids, biomolecular condensates have a spherical shape and can fuse into a single large droplet upon contact with each other. Moreover, compartmentalized condensates are able to dynamically exchange components with the surrounding cytoplasm or nucleoplasm and this can selectively accelerate or inhibit biological reactions (Phair and Misteli, 2000; Snaar et al., 2000). Therefore, LLPS is increasingly being recognized as a fundamental process in the regulation of cellular activities in time and space.

Notably, earlier research on the aberrant LLPS process and formation of condensates, mainly focused on the molecular basis of specific neurodegenerative diseases such as the Alzheimer disease (AD) and amyotrophic lateral sclerosis (ALS) (Dormann et al., 2010; Wegmann et al., 2018). For instance, it was shown that ALS-derived mutations in RNA-binding proteins (RBPs), such as TIA1, HNRNPA1 and FUS, facilitate abnormal accumulation of the proteins in stress granules (which are cytoplasmic condensates) (Kim H. J. et al., 2013; Mackenzie et al., 2017). It was also suggested that this pathologic aggregation of proteins in ALS patients was associated with an altered LLPS process. In addition, *in vitro* experiments confirmed that these proteins had undergone phase separation and condensed into liquid-like droplets, and that over time they were converted into solid-like aggregates, which is the foundation of age-related diseases (Patel et al., 2015). Due to abundant proof that LLPS-derived protein aggregates are responsible for age-related diseases, the role of LLPS in cancer is also gaining increasing attention. For example, RNA *N*^6^-methyladenosine (m^6^A), one of the most prevalent epigenetic modifications on mRNAs, was reported to be associated with diverse cancer biological activities (Ma et al., 2019). Moreover, recent studies showed that m^6^A-modified mRNAs can interact with its binding motifs and phase separate into compartmentalized condensates, leading to altered mRNA expression (Gao et al., 2019). Therefore, given the indispensable role of m^6^A in cancer biology, it is likely that LLPS participates in m^6^A-relavent tumor occurrence and development. In addition to participation in epigenetic modifications, LLPS is also involved in a wide range of cellular activities, such as transcriptional regulation, maintenance of genome stability, and signaling transduction, potentially contributing to tumorigenesis and tumor development.

Therefore, the current article gives a comprehensive review of existing research on LLPS, including a basic description of the process, its known role in cancer and the biological activities in which LLPS is involved that are potentially implicated in cancer. This review also gives a simplified description of the biophysics and biochemical mechanisms underlying LLPS, readers interested in details will be referred to the published literature (Alberti et al., 2019; Dignon et al., 2020).

## Molecular-Level Rules and Components of LLPS

**FIGURE 1:**
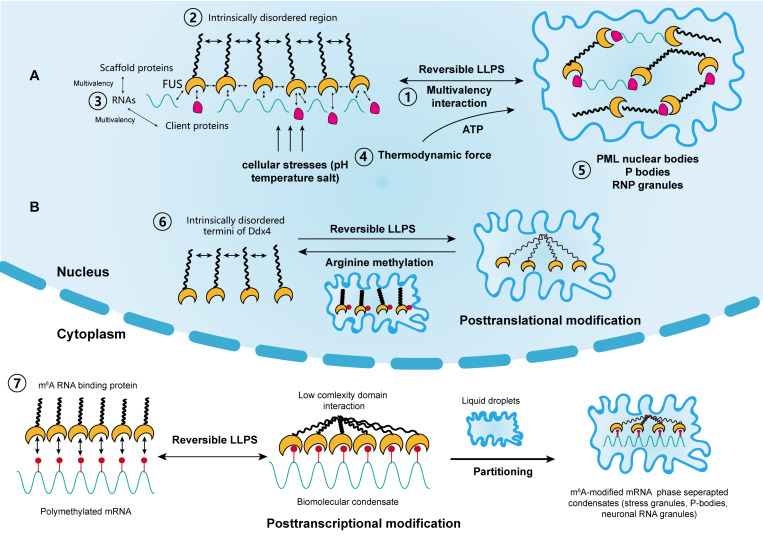
The driving forces and regulation of liquid–liquid phase separation (LLPS) in cellular activity. **(A)** Scaffold proteins, such as FUS, concentrate low-valency client proteins through multivalent interactions, which is key for driving LLPS. The intrinsically disordered regions in some scaffolds also promote this process. Moreover, RNAs can further promote this process through interactions with RNA-binding regions. The thermodynamic force as well as cellular stressors, such as pH, temperature, and salt, may also support the reversible LLPS process. Classical nuclear structures formed by LLPS include PML bodies, P-bodies, and RNP granules. **(B)** LLPS is regulated by posttranslational and posttranscriptional modifications. Liquid condensates formed by Ddx4 are destabilized by its arginine methylation, whereas interactions between poly m^6^A methylated mRNAs and m^6^A binding proteins promote LLPS. Each step corresponds to its numbering.

Varying degrees of weak non-specific interactions between biomolecules are the driving forces behind phase separation at the molecular level (Shin and Brangwynne, 2017; Youn et al., 2019) (Figure 1A). In living cells, this interaction is represented by protein–protein or RNA–protein multivalency (Li et al., 2012). At a given temperature, phase separation occurs above a saturated concentration, beyond which weak transient interactions between biomolecules are stronger than the unfavorable entropy of demixing (Banani et al., 2017; Wang et al., 2018). Therefore, large biomolecules separate into two phases above this concentration threshold, one of which is dilute and the other is highly condensed. For biomolecules in cellular solutions, both phases are typically in a liquid state; thus, the process is termed LLPS (Youn et al., 2019). Furthermore, the probability of a solution to undergo LLPS is not only determined by its concentration and molecular multivalency, but also by environmental conditions such as temperature, salt, and pH (Riback et al., 2017). Additionally, the variety of physical determinants for LLPS further indicate that it can occur due to cellular stressors, like high temperature and hypoxia, which are common inducers for tumorigenesis (Riback et al., 2017).

Proteins and nucleic acids (RNA and DNA) are major components and mediators of LLPS. They can be altered by their biophysical properties as well as phase separation behaviors to form the highly multicomponent systems in condensates (Banani et al., 2016). The initially established multivalent interactions for phase behaviors occur between proteins containing repeats of the SRC homology 3 (SH3) domains and its binding proline-rich motifs (PRMs) (Li et al., 2012). In *in vitro* experiments, Li et al. (2012) demonstrated that these multi-valent, folded SH3/RPM domains are the drivers of phase separation. Additionally, the formation of liquid droplets was more evident at a higher SH3 concentration and valency (Li et al., 2012). Furthermore, Li et al. (2012) constructed a system containing nephrin, neural Wiskott–Aldrich syndrome protein (N-WASP) and NCK; they showed (*in vivo*) that the repeated SH2/SH3 domains from Nck and N-WASP, along with the phosphorylation of nephrin promoted the formation of liquid condensates through LLPS, which initiated the assembly of actins mediated by Arp2/3. Notably, nephrin plays a critical role in regulating the formation of glomerular filtration barrier, through actin assembly in kidney podocytes (Rohatgi et al., 2001; Jones et al., 2006). In addition, proteins that exhibit the same repetitive domains are often thought to be involved in the formation of signaling complexes, as in the case of T cell receptor (TCR) clusters in transmembrane signaling (Su et al., 2016). The process will be discussed in detail in subsequent paragraphs.

Proteins are the most common components of condensates, and can be classified into scaffolds and clients according to their functions (Banani et al., 2016). Scaffold proteins drive LLPS and maintain the integrity of condensates, whereas client proteins are low-valency molecules, which are concentrated by scaffolds during LLPS and dynamically exchange with the surrounding cytoplasm and nucleoplasm. Notably, members of the FUS family are typical scaffold proteins. They drive the accumulation of gel-like condensates, leading to the progression of ALS and age-related diseases (Crozat et al., 1993; Patel et al., 2015). Other scaffold proteins include HNRNPA1, HNRNPA2, TAF15, and ESWR1 (Lin et al., 2015; Ryan et al., 2018). In addition, scaffold proteins and RNP granules usually possess intrinsically disordered regions (IDRs). Typically, these regions are enriched with charged amino acids, which constitute the backbone of different kinds of charged interactions between RNA and proteins (Castello et al., 2012; Nott et al., 2015; Uversky, 2017). Moreover, compared with structured proteins with highly folded sequences, IDRs have low-sequence complexity domains (LCDs). These structural features make them energetically favorable and facilitate the occurrence of LLPS (Burke et al., 2015; Elbaum-Garfinkle et al., 2015; Molliex et al., 2015; Conicella et al., 2016).

RNA is another significant component of condensates. It acts in concert with proteins to regulate LLPS in living cells (Zhang et al., 2015). Biomolecular condensates in the nucleus, such as the nucleolus, paraspeckles, and RNP granules all contain large amounts of both proteins and RNA as a result of LLPS (Berry et al., 2015). Additionally, RNA is an ideal scaffold element for its single-stranded, multivalent, and flexible structures. For example, a reduction in RNA concentration or a genetic depletion of RNA-binding in the nucleus accelerates LLPS, leading to the accumulation of cytotoxic gel-like FUS and TDP43 protein aggregates (Maharana et al., 2018). In contrast, a high RNA/protein ratio inhibits LLPS and the formation of biomolecular condensates. It was also suggested that RNA can buffer the aberrant protein aggregates arising from LLPS. Moreover, RBPs, such as RNA recognition motifs, were reported to play a role in RNA stability and metabolism. These proteins were shown to be responsible for interactions with RNAs during LLPS, which leads to the formation of nuclear RNP bodies, P-bodies, and stress granules (Clery et al., 2008). Furthermore, Molliex et al. (2015) reported that LLPS, mediated by a low-complexity sequence of HNRNPA1, was facilitated by RNA recognition motifs in the presence of RNA, during the persistent assembly of stress granules. This process was independent of IDRs and highlighted the significance of RNA in LLPS (Molliex et al., 2015). In most cases, proteins that contain both IDRs and RNA recognition motifs interact with RNAs to promote LLPS synergistically (Castello et al., 2012). This is now considered as an RNA-induced lowering of saturation concentration for LLPS, which is a prevalent effect on proteins (Lin et al., 2015; Molliex et al., 2015; Saha et al., 2016).

## Regulation of LLPS in Cellular Activities

It remains unclear how the aging-related accumulation of protein aggregates, such as HNRNPA1 TIA1, and TAU progresses from liquid droplets into gel-like or solid-like particles and eventually nucleate into amyloid fibers. Mechanistically, the production of condensates by LLPS is reversible, although this reversibility is compromised over time within a pathological context (Lin et al., 2015; Zhang et al., 2015). Recent studies suggested that the solid-like state of these proteins is likely to be regulated by transcriptional activities that require the participation of energetic substances, such as adenosine triphosphate (ATP) (Jain et al., 2016; Mugler et al., 2016; Patel et al., 2017). Intriguingly, several studies have shown that nucleolar subcompartments and those within RNP granules have an apparent nucleolar viscosity, which is highly dependent on ATP, and that the fluidity of stress granules significantly decreases in the absence of ATP (Brangwynne et al., 2011; Feric et al., 2016; Jain et al., 2016). Moreover, it was reported that nucleoli undergo LLPS and preferentially assemble in the organizing regions of transcriptionally active rRNAs, in processes driven by thermodynamics (Berry et al., 2015). In addition, nucleolar components and structures show substantial changes after inhibiting transcription upon nucleolar condensation (Shav-Tal et al., 2005). Therefore, it is hypothesized that both the irreversible transformation of condensates and the changes in the viscosity of nucleolar subcompartments are driven by thermodynamic forces, which are modulated by transcriptional activities. The assumption is consistent with the findings from previous studies which showed that RNA can co-transcriptionally drive the assembly or nucleation of nuclear organelles such as the nucleolus, paraspeckles, and stress granules (Mao et al., 2011b; Shevtsov and Dundr, 2011). However, the thermodynamic parameters modulated by RNA transcription do not fundamentally alter the passive LLPS process. More specific experimental methods that include parameters such as temperature and pH could further elucidate the determinants of LLPS in biological activities. Furthermore, targeting specific RNA transcription or ATPase may be a new therapeutic strategy that can reverse the irreversible process of protein accumulation in cancer and aging diseases. Nonetheless, global ATP depletion or inhibition of transcription can influence normal cell function. Considering their multifaceted roles in cellular activities, targeted RNA interference or specific ATPase abrogation might be promising strategies for managing both diseases.

In addition, the metastability of biomolecular condensates highlights that these molecular structures, represented by proteins and RNAs, likely didn’t reach thermodynamic equilibrium in most cases. Additionally, cells use functional modification as another regulatory mechanism to control the dynamics, composition and reversibility of phase separation, including posttranslational modification (PTM) and posttranscriptional modification (Figure 1B). Representatives of PTM in LLPS include phosphorylation and arginine methylation, which tend to facilitate or impair the formation of condensates by altering the valence of scaffold and client proteins. For example, it was reported that arginine methylation of Ddx4, whose IDRs condense to form condensates through LLPS, could destabilize the condensates and attenuate LLPS by altering its electrostatic interactions (Nott et al., 2015). Moreover, arginine methylation occurs within the nucleus of cervical cancer cells. In contrast, phosphorylation can either promote or impair the formation of condensates. For instance, synapsin, is a neurotransmitter-containing synaptic vesicle (SV) that forms through LLPS. It was previously shown to rapidly dissolve upon phosphorylation by Ca^2+^/calmodulin-dependent kinase II to trigger the release of neurotransmitters (Milovanovic et al., 2018). Similar phenomena were observed in the low-sequence-complexity domains of FUS and other amyloid proteins (Monahan et al., 2017; Carpenter et al., 2018). Intriguingly, phosphorylation of the microtubule-binding domain of the tau protein instead altered its valency and facilitated the formation of liquid condensates, eventually leading to the aggregation of amyloid tangles (Ambadipudi et al., 2017). The modulatory effect of PTM on LLPS therefore shows its potential in cancer treatment, by tampering with the interaction between small molecules and target protein motifs.

Posttranscriptional modification is a newly identified mechanism of regulating LLPS and research has mainly focused on RNA m^6^A (Gao et al., 2019; Ries et al., 2019; Liu et al., 2020; Wang J. et al., 2020) (Figure 1B). m^6^A methylation is the most prevalent epigenetic modification at the posttranscriptional level and is widely involved in numerous cellular processes. Ries et al. (2019) reported that LLPS is significantly facilitated by mRNAs containing multiple m^6^A-modified domains through the regulation of m^6^A readers. Moreover, polymethylated mRNA can serve as a multivalent scaffold for the binding of YTHDFs (the RBPs in the m^6^A reader family), and simultaneously juxtapose their low-complexity domain to promote LLPS. Eventually, the mRNA–YTHDF combinants phase separate into compartmentalized droplets, forming liquid condensates such as stress granules and P-bodies, resulting in altered mRNA translation and degradation. Due to the prevalence of aberrant m^6^A modification in tumor biology, therapies targeting m^6^A to modulate LLPS and eventually, the expression of mRNA may have considerable potential (He et al., 2019).

## The Emerging Role of LLPS in Cancer

Over the past decades, there has been great progress in understanding of the malignant behaviors of tumors, and this is represented by the proposal on the hallmarks of cancer (Hanahan and Weinberg, 2011). Notably, during the multistep development of tumors, cancer cells acquire six biological capabilities that allow them to evolve progressively to a malignant state. These include limitless proliferation, avoiding inhibition of growth, resistance to death, replicative immortality, induction of angiogenesis, invasiveness and distant metastasis, (Hanahan and Weinberg, 2000). It was confirmed that cancer mutations typically underpin the acquisition of these hallmarks, or they interfere with the functions of inhibitors of these hallmarks. However, it is still unclear why certain genetic mutations cause cancer. Therefore, the emergence of phase separation offers a new basis of interpreting and understanding cancer phenotypes, with potentially novel therapeutic avenues.

### P53 Protein Aggregation: Question to Answer

**FIGURE 2:**
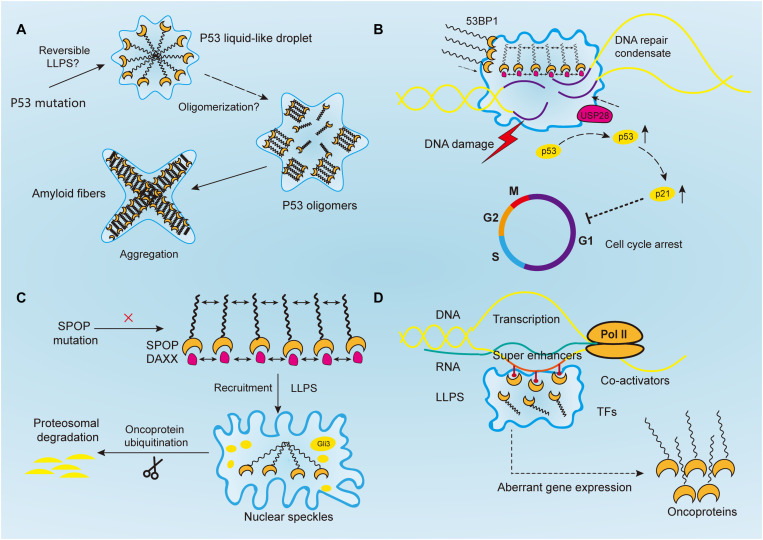
Aberrantly regulated liquid–liquid phase separation (LLPS) in cancer. **(A)** Tumor suppressor p53 forms liquid-like droplet upon mutations, and it self-associates into oligomers through oligomerization. Consequently, p53 oligomers irreversibly aggregate into amyloid fibers. However, it is still unclear whether p53 undergoes reversible LLPS to form droplet. **(B)** Upon DNA damage, 53BP1 is recruited and assembles at the DNA damaged sites through phase separation. The phase separated 53BP1 condensates dynamically recruit and stabilize downstream proteins such as p53 and its co-activator USP28, leading to the overexpression of p53 target genes such as p21 and consequently arrest of cell cycle. **(C)** Highly ordered SPOP polymers and their binding protein substrates, such as DAXX, undergo LLPS and are recruited toward nuclear speckles, where SPOP oligomers facilitates the ubiquitination of substrates such as Gli3 and other oncoproteins to inhibit tumorigenesis. In cancers, this function is abrogated by SPOP mutation. **(D)** Transcriptional regulators, including super-enhancers, co-activators, transcription factors, and RNA polymerase II, all undergo a phase separation that promotes cancer transcriptional activities, resulting in the aberrant expression of oncogenes and facilitating tumor progression.

As in the case of neurodegeneration, disease-linked genetic mutations in RBPs promoted the accumulation of their respective proteins, which forms membraneless-organelles in the cytoplasm or nucleus through reversible phase separation (Kim H. J. et al., 2013; Patel et al., 2015; Conicella et al., 2016; Mackenzie et al., 2017). A higher concentration of these proteins in condensates shifted the phase equilibrium toward a highly condensed state and simultaneously promoted the formation of irreversible protein aggregates (Patel et al., 2015; Wegmann et al., 2018). This irreversible liquid-to-solid transition corresponds to the formation of amyloid fibrils, which underlies the development of aging diseases (Shin and Brangwynne, 2017). However, the same mechanism is yet to be proven in cancer mutations. For instance, TP53, one of the most studied tumor suppressor genes, plays a critical role in maintaining genomic stability. It protects cells against tumorigenesis by inducing cell cycle arrest and binding to its target DNA sequence to initiate DNA repair or apoptosis (Silva et al., 2014; Eischen, 2016; Mantovani et al., 2019) (Figure 2A). Over 50% of human cancers exhibit TP53 gene mutations (Muller and Vousden, 2013). Previous studies reported that p53 can be uptaken into nuclear bodies such as the Cajal and PML bodies under conditions of stress responses (Fogal et al., 2000; Guo et al., 2000; Cioce and Lamond, 2005). In prostate cancer, p53, along with other proteins such as MYC and CDKN2D, accumulates within significantly enlarged cancer cell nucleoli (Dang and Lee, 1989; Wsierska-Gadek and Horky, 2003; Fischer et al., 2004; Pedrote et al., 2018). Moreover, mutant p53 protein is known to form amyloid-like aggregates, which abrogate its antitumor functions (Ghosh et al., 2017). It was also reported that mutant p53 aggregates faster than the wild-type, and the state transition is largely attributed to the p53 DNA binding domain, which has an amyloidogenic sequence and is likely to associate into solid-like fibrils (Lee et al., 2003; Ano Bom et al., 2012; Ghosh et al., 2017). Aggregated species of mutant p53, or in another word, oligomerization of p53 mutants, are highly expressed in cancer cells, and accumulate as amyloid oligomers, which are associated with malignant phenotypes such as chemoresistance and tumor growth (Melo Dos Santos et al., 2019; Pedrote et al., 2020; Zhang et al., 2020). Although oligomerization and phase separation are often coupled, they do represent distinct process, and can be experimentally separated. Therefore, notwithstanding the seemingly alike outcomes of aging-associated amyloidogenesis and p53 aggregation, the process they resulted from could be distinct.

Intriguingly, recent studies reported potential condensation-like behaviors of mutant p53 that exhibited a number of unique features, distinct from LLPS-originated liquid droplets. For instance, Yang et al. (2020) reported that p53 R248Q, one of the p53 mutants that strongly predicts poor outcomes in ovarian cancer patients, forms condensate-like clusters by destabilizing the structure of its core domain instead of interacting through its disordered regions. Moreover, the formed p53 R248Q clusters may lead to the misfolding and irreversible aggregation of p53 itself, and consequently amyloid fibrils. The condensate-like state was also observed in fluorescently tagged structural mutant p53 in osteosarcoma cells, and they were independent of PML or Cajal bodies (Lemos et al., 2020). Another study reported that p53 itself was found to form liquid-like droplets *in vitro*, at a neutral and slightly acidic pH environment, and at low salt concentrations (Kamagata et al., 2020). Although low-complexity domains that frequently being found in phase-separated proteins was not identified in p53, the multivalent electrostatic interactions between the C-terminal and N-terminal domains of p53 mutant were proven to be the key drivers for droplet formation (Kamagata et al., 2020). Additionally, this process was found to be regulated by molecular crowding agents, DNA, and PTM, while the effect of RNA on droplet formation is still unclear (Kamagata et al., 2020). Given the above studies, we presumed that the pathologic cancer-associated p53 aggregation may proceed via a functional step of LLPS, which explains why it shares similarities with LLPS yet exhibit distinguishable characteristics. Nevertheless, more studies are needed to ascertain the roles of p53 in cellular conditions, to determine its relationship with LLPS and amyloid formation, and to elucidate its possible involvement in the formation of membraneless organelles such as the Cajal and PML bodies.

### Maintenance of Genome Stability

Genetic mutations are usually accompanied by the disruption of DNA damage response (DDR) and DNA repair, as exemplified in p53. Recent studies indicate that DDR can induce the formation of transient repair condensates at the sites of DNA damage to concentrate repair proteins and activate repair signaling (Jackson and Bartek, 2009; Kim et al., 2020). Poly(ADP-ribose) polymerase 1 (PARP1), the founding member of the PARP family, plays a crucial role in cancer biology, including chromatin remodeling, replication, transcription, and most importantly, DNA repair and genome maintenance (Ray Chaudhuri and Nussenzweig, 2017; Hanzlikova and Caldecott, 2019; Kim et al., 2020). Early studies also showed that it was overexpressed in a variety of cancers (Rouleau et al., 2010). PARPs, such as PARP1, that are capable of synthesizing negatively charged polymers of PAR chains are called “writers” of poly(ADP-ribosyl)ation (PARylation) (Kamaletdinova et al., 2019). PARylation is thought to regulate the biochemical properties, assembly and catalyzation of target proteins upon DNA damage in a spatio-temporal confined manner (Kim et al., 2020; Pahi et al., 2020). Recently, Altmeyer et al. (2015) demonstrated that the formation of PAR chains, its nucleic-acid like properties, and its multivalent anionic nature are prerequisite to trigger the assembly of proteins containing IDRs at the damaged DNA sites, and thereby initiate liquid demixing. Consequently, a transient and reversible intracellular compartmentalization is achieved in response to DNA damage, via selective recruitment of LCD-containing FET proteins such as FUS, TAF15, and EWS (Altmeyer et al., 2015). Moreover, the LLPS behavior driven by electrostatic interactions between the positively charged LCDs and negatively charged PARs is amplified by prion-like protein domains, facilitates DNA repair (Altmeyer et al., 2015). In fact, PARP inhibitors have been widely used in clinic as monotherapeutic agents to block single-strand DNA (ssDNA) repair thereby inducing the synthetic lethality in cancers including ovarian, breast, and pancreatic cancer (Jiang et al., 2019; Slade, 2020; Zhu et al., 2020). With more functional complexity of PARPs revealed, such as its role in LLPS, higher efficacy of clinical medicine may be achieved through combinational treatment method targeting both LLPS and DNA DDR.

Following PAR signaling studies, p53-binding protein 1 (53BP1) has also received attention for its important role in maintaining genomic stability (Figure 2B). By binding with p53, 53BP1 directly regulates the stability of p53 and affects the expression of p53 target genes, and it has been reported to regulate tumor cell behaviors in a variety of malignancies such as esophageal, colorectal, and breast cancers (Misteli and Soutoglou, 2009; Bi et al., 2015; Mirza-Aghazadeh-Attari et al., 2019; Yang et al., 2019; Schochter et al., 2020). And reduction of 53BP1 expression was reported to be associated with cell cycle arrest in esophageal cancer cells (Yang et al., 2019). Previous studies have reported that 53BP1, as a regulator of the repair of DNA double-strand breaks (DSBs), facilitates formation of chromatin domains around the damaged DNA, where downstream effectors are simultaneously recruited to shield the DNA lesions against nucleolytic reactions (Panier and Boulton, 2014; Mirza-Aghazadeh-Attari et al., 2019). However, the mechanism of how repair proteins assemble in time and space at the DNA damaged sites was not revealed until recently. Kilic et al. (2019) confirmed the liquid droplet-like properties of 53BP1 assembly and proved their phase separated behaviors, which was mainly contributed by its C-terminal regions that highly enriched with tyrosine and arginine and oligomerization domains. Addition of chemical 1, 6-hexanediol, that disrupts hydrophobic interactions reversed LLPS processes and the assembly of 53BP1 (Kilic et al., 2019). Additionally, Pessina et al. (2019) demonstrated that long non-coding RNA synthesized at DSB can also drive the formation of 53BP1 assemblies through LLPS, and the process was halted upon transcription inhibition. Notably, the phase separated 53BP1 assemblies for repairing DNA damage dynamically recruit and stabilize downstream proteins such as p53 and its co-activator USP28 (Kilic et al., 2019). When the formation of assembly is damaged, p53 stability is consequently impaired, and so as its target gene p21, leading to cell cycle arrest (Kilic et al., 2019). Although corresponding inhibitors of 53BP1 has not been developed yet, its role in DNA damage repair by means of LLPS makes it promising target for inducing cancer cell death. Given their similar phase separated behaviors, 53BP1 might be used to sensitize chemoresistance cancer cells in combination with PARP inhibitors (D’Andrea, 2018).

### SPOP

The speckle-type pox virus and zinc finger (POZ) domain protein (SPOP) is a substrate adaptor that pairs with the cullin3-RING ubiquitin ligase (CRL3). SPOP controls the ubiquitination and subsequent proteasomal degradation of various protein substrates such as the death domain-associated protein (DAXX), androgen receptor (AR), epigenetic regulators and hormone signaling effectors, therefore is involved in diverse cellular activities, including cell cycle control, epigenetic modification and hormone signaling (Mani, 2014). Mutation of SPOP and the resulting dysregulation of ubiquitin-proteasome pathways play an important role in the pathogenesis and progression of endometrial and prostate cancers, whereas its overexpression and mislocalization are correlated with kidney cancer (Li G. et al., 2014; Geng et al., 2017; Cuneo and Mittag, 2019; Wang Z. et al., 2020). SPOP is most frequently mutated in prostate cancer, and across 21 various types of cancers (Lawrence et al., 2014). In prostate cancer, SPOP mutation abrogates its interaction with the SRC-3 protein and subsequent regulation on AR transcription, leading to tumor progression and resistance to androgen deprivation therapies (Geng et al., 2013, 2017).

Human SPOP is mainly composed of three domains, in which the N-terminal meprin and TRAF-C homology (MATH) domain is the core domain responsible for recognizing protein substrates, while the other two synergistically promote the self-association of SPOP into linear, high-order oligomers (Marzahn et al., 2016). The size distribution of oligomers is highly dependent on SPOP concentration (Marzahn et al., 2016). Previous studies have proved that wild-type SPOP mainly localizes onto membraneless liquid nuclear speckles, where the interchromatin clusters are critically involved in mRNA maturation, DNA repair, RNA metabolism, and chromatin organization (Figure 2C) (Nagai et al., 1997; Galganski et al., 2017). The highly ordered SPOP oligomer can recruit substrates such as Gli3 and other oncoproteins to CRL3 for ubiquitination and subsequent proteasomal degradation, which mechanistically reduce tumorigenesis (Zhang et al., 2006, 2009; Cheng et al., 2018). However, the mechanism about how SPOP recruits oncogenic substrates to nuclear speckles remains elusive. Recently, Bouchard et al. (2018) reported that SPOP localizes onto liquid nuclear organelles through phase separation with DAXX, which contains multiple SPOP-binding motifs in intrinsically disordered domains. Moreover, they proved oligomerization of SPOP instead of its monomeric state can promote LLPS (Bouchard et al., 2018). In other words, the multivalent interactions between highly ordered SPOP oligomers and its binding substrate proteins can promote LLPS and their co-localization. Additionally, prostate cancer-associated SPOP mutants inhibit substrate ubiquitination by interfering with the LLPS process (Bouchard et al., 2018). Therefore, it is concluded that SPOP mutations can lead to the accumulation of oncogenic proteins, which in turn disrupts the formation of phase-separated condensates that regulate ubiquitin-dependent proteostasis. This may provide a theory basis for prostate cancer treatment that involves relocalizing SPOP to nuclear speckles by decreasing its saturation concentration or inventing substrate motifs compatible with SPOP, thereby restoring LLPS and the ubiquitination balance to inhibit tumor progression.

### Transcription and Super-Enhancers

The development of cancer involves the aberrant regulation of downstream transcriptional networks, following the mutation of driver genes. A variety of cancer phenotypes including tumor growth, invasion, chemoresistance and distant metastasis, are driven by deregulated transcription activities (Bradner et al., 2017). Notably, RNA transcription requires the participation of RNA polymerases (Pol) and transcription factors (TFs), which are both likely to be regulated by phase separation (Figure 2D). For instance, the carboxy-terminal domain (CTD) of RNA Pol II is a low-complexity domain with an IDR, which is vital for mRNA processing and transcription. It was previously observed that reducing the length of CTD led to a decrease in Pol II clustering and the recruitment of transcriptional apparatus, whereas extension of the CTD resulted in the opposite effect (Boehning et al., 2018). Mechanistically, CTD droplets can undergo phase separation to incorporate Pol II at gene promoters, and phosphorylation of CTD releases the Pol II from clusters for transcription elongation (Boehning et al., 2018). A similar process was shown to occur in TFs. Moreover, the activation domains from the TFs OCT4 and GCN4, when driven by weak multivalent interactions are phase separated into liquid droplets. Estrogen receptor, a ligand-dependent TF, also undergo LLPS for gene activation (Boija et al., 2018). Most activation domains on TFs contain intrinsically disordered low-complexity sequence domains. Additionally, the interactions between low-complexity sequence domains underlie LLPS and the subsequent stability of DNA binding, polymerase recruitment, and transcriptional activation (Chong et al., 2018). Moreover, phase separation of TFs has been reported to be implicated in the regulation of aberrant gene expression in cancers, as exemplified by EWS and FLI1 in Ewing’s sarcoma, suggesting that disturbing the interactions of specific TF domains is a potential drug therapy (Chong et al., 2018).

Additionally, the emergence of LLPS provides a novel framework for understanding the molecular mechanisms of underlying dysregulated transcription, mediated by oncogenes such as MYC, whose regulatory basis is yet to be revealed. Recent researches have focused on a bunch of gene clusters called ‘super-enhancers,’ which are essential for regulating MYC expression levels in hematological tumors and colon cancer cells (Bahr et al., 2018; Scholz et al., 2019). Literally, super-enhancers are clusters of transcriptional enhancers or regulatory elements that mainly consist of master TFs and mediators (Hnisz et al., 2013; Whyte et al., 2013). Compared to conventional TFs, super-enhancers drive more robust expression of genes that prominently modulate cellular functions and determine cell identity. Therefore, they are expected to play an important role in cancer (Sengupta and George, 2017). Moreover, existing evidence shows that the high-density transcriptional apparatus of super-enhancers has the properties of biomolecular condensates, such as a sharp phase transition, the dynamic exchange of substances, and collapsing with the depletion of condensation, indicating the involvement of LLPS in the clustering of super-enhancers (Loven et al., 2013; Kwiatkowski et al., 2014; Hnisz et al., 2015; Proudhon et al., 2016; Sengupta and George, 2017). Recently, Sabari et al. (2018) reported that the transcriptional mediator co-activators BRD4 and MED1 are enriched by super-enhancers and can phase separate into compartmentalized condensates and concentrate the transcriptional apparatus to control gene expression. Therefore, we speculated that IDR-driven phase separation of super-enhancers or other transcriptional apparatus is a general mechanism for gene activation at the promoters by TFs.

With better understanding of the role of LLPS in transcriptional activities, Hnisz et al. (2017) proposed a phase separation model that can predict key determinants in transcriptional regulation. Upon epigenetic or chemical modifications, the components of enhancers, including multivalent proteins and nucleic acids (RNA and DNA), can form chain-like structures, and the residues could potentially interact with other chains, thus building cross-links (Hnisz et al., 2017). After a cascade of reactions, molecules within this system, such as master TFs, mediator co-activators, chromatin regulators, and RNA polymerases, can achieve a dynamic balance and phase separate into droplets, which cooperatively regulate transcriptional activities (Hnisz et al., 2017). Based on this theory, some mechanisms of transcriptional activities aberrantly regulated by enhancers in cancer can be partially explained. For instance, Mansour et al. (2014) demonstrated in T cell leukemias that the mutation of TAL1 oncogene enhancer can create binding motifs for master TF MYB, which recruits additional parts of the transcriptional apparatus, such as co-activator acetyltransferases CBP, as well as critical components of leukemic transcriptional complex, including GATA-3, RUNX1, and TAL1. Namely, the insertion of a single TF MYB has the potential to bind other co-factors at the transcriptional domain, leading to the initiation of super-enhancers upstream for the activation of TAL1 oncogene, and consequently promote leukemogenic progress (Mansour et al., 2014). From our perspective, the step-wise manner of the recruitment of TAL1 super-enhancer complex, and their spatio-temporal proximity on transcriptional domains correspond to certain characteristics of the formation of biomolecular condensates, indicating a potential role of LLPS in this process. In addition, the genetic deletion of enhancers within super-enhancers can reduce the recruitment of other co-factors, and lead to the collapse of the entire super-enhancer (Mansour et al., 2014). A reversible state of this process may further confirm the association between LLPS and transcriptional regulation in cancer. Thus, the LLPS model proposed by Hnisz et al. (2017) might be used for the prediction of potential enhancers aberrantly activated in cancers for downstream transcriptional regulation at promoters, and for therapies targeting specific driver genes such as MYB or MYC to block downstream gene expression and protein translation.

## Transmembrane Signaling Transduction Regulated by LLPS: Implications in Cancer

Cancer cells are continually exposed to aberrant external signals that are accepted by transmembrane receptors, which in turn initiate diverse intracellular signaling cascades, resulting in the malignant behaviors of cancer cells. Sustaining of proliferative signaling, for example, one of the most fundamental traits of cancer cells, is associated with classical cancerous signals such as the PI3K/Akt, JAK/STAT, and NF-κB signaling pathways, all of which involve membrane receptors assembling into two-dimensional clusters with transmitted molecules. Intriguingly, recent studies suggested that LLPS might be an essential mechanism for the high-order assembly of these receptors driven by weak, multivalent interactions. Therefore, we uncover two relevant signaling pathways not only play important roles in tumorigenesis but also involve LLPS.

### LLPS in Immune-Relevant Signaling Pathways

**FIGURE 3:**
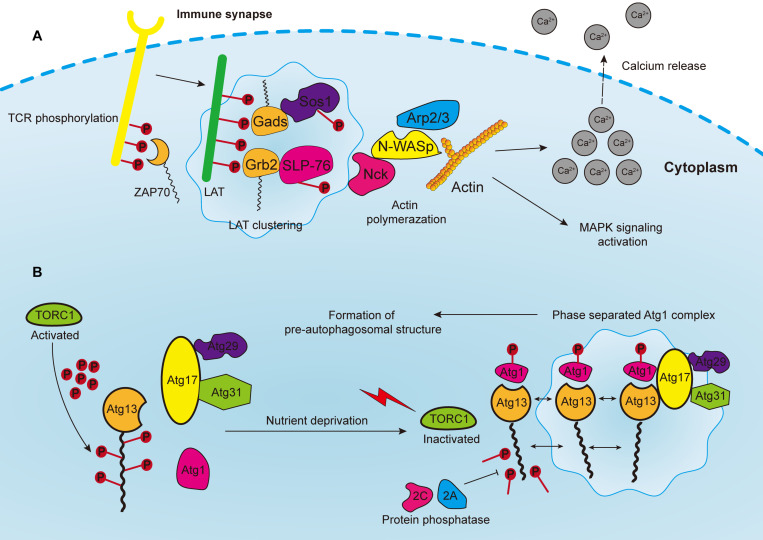
**(A)** Phosphorylated T cell receptors (TCRs) recruit and activate cytoplasmic tyrosine kinase ZAP70, which in turn phosphorylates the linker for the activation of T cells (LAT). LAT drives the formation of clusters enriched with downstream proteins, such as Gads and Grb2, which recruit adaptor proteins, such as Sos1 and SLP76. Phosphorylated Sos1 and SLP76 initiate the recruitment of actin effectors such as WASP, Nck, and Arp2/3 complex for polymerization of actin filaments, consequently leading to calcium release, MAPK signaling activation, and the formation of an immune synapse. **(B)** In yeast cells, the Atg1 complex consists of five subunits including Atg1, Atg13, Atg17, Atg29, and Atg31, which are abundant with IDRs for subsequent phase separation. The Atg13 is highly phosphorylated by TORC1 under nutrient replete conditions, leading to the block of Atg1 complex formation. Upon nutrient deprivation, TORC1 is inactivated, and Atg13 is therefore dephosphorylated by protein phosphatases 2C and 2A, whereas Atg1 is auto-phosphorylated. Atg13 serves as a scaffold protein to bind with Atg1 and Atg17-Atg29-Atg31 to promote the phase separation of Atg1 complex, and consequently the formation of pre-autophagosomal structure.

The assembly of signaling clusters on the membranes of immune cells has been observed for several decades. Previous studies reported that receptors on both T and B cells are assembled on membranes in response to external stimulation (Schumacher, 2002; Bankovich and Garcia, 2003; Gold and Reth, 2019). T cell antigen receptor (TCR) signaling is a well-studied system to elucidate the formation of transmembrane clusters (Figure 3A). Notably, linker for the activation of T cells (LAT) is a transmembrane adaptor protein that centrally modulates most downstream signals of the TCR at immune synapses [the interface between bound immune cells, such as when T cells bind with antigen-presenting cell (APC)] (Dustin and Choudhuri, 2016). Upon TCR activation, the tyrosine residues of LAT are phosphorylated by the membrane kinase ZAP70 (Zhang et al., 1998). These phosphorylated tyrosine residues are in turn necessary for attracting the multivalent Src Homology protein family members including Grb2, Gads and PLC-γ, and subsequent formation of membranous clusters and signal transduction (Samelson, 2002; Balagopalan et al., 2015). Thereafter, the PRMs of the adaptor proteins, such as Sos1 and SLP76, bind accordingly to the multivalent domains of Grb2 and Gads, and then initiate the recruitment of actin effectors such as WASP, Nck, and the Arp2/3 complex for the polymerization of actin filaments (Kumari et al., 2015; Huang et al., 2016). Consequently, these actin regulators and kinases induce TCR activation, calcium release, actin assembly and stimulation of MAPK signaling.

Bunnell et al. (2002) have reported that if TCR binds to stimulatory antibodies, clusters enriched with SLP-76, LAT, ZAP-70 and other signaling partners could form, leading to the generation of an immune synapse. Moreover, these clusters are formed without lipid membrane structures, and the components of clusters could be dynamically exchanged with the surroundings. However, it was not until 2016, that the role of LLPS in this process was uncovered (Balagopalan et al., 2015; Su et al., 2016). Su et al. (2016) biochemically reconstituted an *in vitro* TCR signaling system and revealed that the biochemical force-multivalency between molecules in LAT clusters interacted and resulted in phase separation. Additionally, CD45, one of the transmembrane phosphatases opposing TCR phosphorylation, was excluded from the LLPS-derived clusters, indicating that LLPS produces a specialized chemical environment by selectively concentrating molecules in clusters. Therefore, it is reasonable to speculate that other signaling pathways, or more specifically, the activation of other immune cells, employ the same mechanisms since clusters are prevalent on immune cell receptors.

Furthermore, it was assumed that a large proportion of biomolecules in the transmembrane signaling receptors on different T cells can phase separate into clusters to facilitate the transduction of signals and to regulate immune responses in cancers. Based on this assumption, therapies aimed at TCR and LLPS could be generalized to other co-inhibitory receptors on T cells, such as the programmed cell death protein 1 (PD-1), the cytotoxic T lymphocyte antigen 4 (CTLA-4), and T-cell immunoglobulin and mucin domain 3 (TIM-3) (Wei et al., 2018). For instance, CD28, is one of the co-stimulatory factors for T cells, and is mostly co-localized with PD1 clusters upon T cell activation (Hui et al., 2017). In addition, PD1 frequently recruits phosphatase Shp2, after being activated by its ligand PDL1, for the preferential dephosphorylation of CD28 in order to suppress T cell function (Hui et al., 2017). Therefore, T cells are selectively activated for the regulation of downstream signaling. It is also likely that the tumor-killing effects of immunotherapies may be enhanced by reducing the aberrantly clustered checkpoints binding to multivalent T cell adaptor proteins such as Grb2 or LAT through LLPS in ways such as tuning the biochemical multivalences between interactive domains (Brooks et al., 2004).

In addition to the membrane signaling pathways, LLPS also facilitates intracellular signaling transduction. Notably, cyclic GMP-AMP synthase (cGAS) is a cytoplasmic DNA sensor that catalyzes the generation of cyclic GMP-AMP (cGAMP) from guanosine triphosphate and ATP by binding to double-stranded DNA (dsDNA) (Gao et al., 2013; Sun et al., 2013; Ablasser and Chen, 2019). On the other hand, the secondary messenger cGAMP can activate the adaptor protein STING, which in turn induces type I interferon and consequently, an innate immune response. Du and Chen (2018) reported that double-stranded DNA binding with cGAS prominently promoted their phase separation and the formation of condensates, in which activated cGAS changed the multivalence of DNA. Mechanistically, the altered valence of the cGAS–DNA interaction and the increased protein and DNA concentration cooperatively lowered the saturation concentration. Therefore, the aberrant expression of cGAS or other secondary messengers could make them independent of ligands and constantly activate immune signaling pathways, which might be a viable therapeutic target.

### Autophagy-Relevant Signaling Pathways

Autophagy is an evolutionarily conserved catabolic process that requires the formation of double-membrane vesicles known as autophagosomes to capture intracellular wastes, such as cytotoxic proteins and damaged organelles, and deliver it to the lysosome for degradation and recycling. Environmental stress, such as hypoxia and nutrient deprivation, activates autophagy-related genes (ATGs) in order to initiate and enhance intracellular autophagic degradation to fulfill the metabolic and energy demands of cancer cells. Moreover, autophagosome precursor, also known as the pre-autophagosomal structure (PAS) in yeast, is a transient structure modulated by nutrient signals and frequently forms on vacuoles upon starvation (Fujioka et al., 2020). Formation of PAS starts with the assembly of multiple Atg1/ULK complexes, which in turn recruit other ATG proteins by forming a scaffold (Yamamoto et al., 2016). In yeast cells, the Atg1 complex consists of five subunits, namely Atg1, Atg13, Atg17, Atg29, and Atg31, which are abundant with IDRs for subsequent phase separation (Yamamoto et al., 2016; Fujioka et al., 2020) (Figure 3B). Additionally, the activation of Atg1 requires the phosphorylation of its kinase domain at Thr226, which induces its clustering and makes it serve as a client protein. Whereas Atg13 is dephosphorylated by protein phosphatases 2A and 2C, then binds to the distinctive regions of Atg17 to form scaffold droplets, thus facilitating the LLPS of the Atg1 complex and the formation of autophagosome (Fujioka et al., 2014). Although the initiation of autophagy in mammalian and cancer cells remains unclear, important insights may be obtained from this mechanism in yeast cells. For instance, target of rapamycin complex 1 (TORC1), which covers most of the signaling pathways relevant to autophagy, inhibits the formation of autophagosome, and regulates cell metabolism as well as growth (Saxton and Sabatini, 2017; Murugan, 2019). The Atg1/ULK complex is highly phosphorylated by TORC1 under nutrient replete conditions, leading to the block of downstream signaling and inhibition of autophagy (Jung et al., 2010). In addition, rapamycin, which is one of the most widely used immunosuppressors, can effectively inhibit Atg1/ULK phosphorylation by targeting mTORC1 (Li J. et al., 2014). Moreover, rapamycin can block the PI3K/Akt/mTOR pathway to inhibit tumor growth (Dibble and Cantley, 2015). Since rapamycin can promote autophagy, one proposal is to develop synergistic small molecules to accelerate cell autophagy, and to cooperatively induce the autophagic death of cancer cells, thereby enhancing the tumor-killing effect of rapamycin (Kim and Guan, 2015). These small molecules will act as scaffold proteins binding to Atg1/ULK clusters to facilitate their LLPS process.

Additionally, recent studies showed that p62, one of the autophagic receptors targeting autophagosomal cargoes for degradation, can assemble into liquid condensates by binding with ubiquitinated proteins, and it is itself degraded through autophagy (Sun et al., 2018; Zaffagnini et al., 2018). Furthermore, the binding of p62 to the KEAP1 mutant and NRF2 stabilizes NRF2-regulated transcription, which alters cell metabolism and alleviates oxidative stress (Cloer et al., 2018). However, when the role of p62 in selective autophagy is impaired, it accumulates and regulates downstream signaling such as NRF2, mTORC1, and NF-κB. This in turn leads to imbalanced cellular oxidation, nutrition conditions and inflammatory responses, consequently enhancing the progression of cancer (Sanchez-Martin et al., 2019). Therefore, p62 is likely to play an important role in autophagy-relevant cancer signaling, whereas detailed mechanism is needed to be determined in future experiments.

## Conclusion

Tumorigenesis and cancer development are complex processes involving multiple steps, such as gene mutation, alteration of transcription, epigenetic modification, and abnormal protein expression. In addition, all the aberrations in each step collectively result in the occurrence and progression of cancer. Moreover, understanding the roles of LLPS in oncogenic activities, either individually or in combination with other mechanisms, can potentially promote the translation of fundamental research into novel cancer diagnosis and targeted therapies. The biophysical and biochemical interactions between every single biomolecule or their constituent structures underlies the basic principles of LLPS. Mechanistically, thermodynamic forces derived from various cellular activities, such as RNA transcription, PTM, and m^6^A modification, cooperatively regulate phase separation. The maintenance of genome stability is also actively accompanied by LLPS. Since genetic mutations underlying the acquisition of cancer hallmarks are usually accompanied by genome instability, restoring LLPS may be helpful to inhibit tumorigenesis. Deregulated transcriptional activities might upregulate oncoproteins and lead to tumor onset, which is closely related with LLPS. Furthermore, master transcriptional regulators, such as super-enhancers, are crucial for the assembly of co-activators, a process in which LLPS has an important role. Therefore, it could be useful to interfere with the aberrant activation of transcription in cancers by targeting the biomolecules that trigger LLPS in condensates. Additionally, signal transduction, though not directly linked with cancer through LLPS, might be a potential target for cancer therapy. Existing evidence has not only demonstrated its regulatory effects on innate immune activation but also its significance in autophagy, both of which are key drivers for cancer progression. Overall, addressing the functionalities of intracellular biomolecules that are associated with higher-order organization, such as proteins, RNAs and DNAs, has deepened our understanding of the biological behavior of cancer. Nevertheless, more effort should be directed toward the functional exploration of LLPS in cancer because many problems remain unsolved.

## Author Contributions

JL wrote the first draft of the manuscript. JQ and ZX conceived the idea of the manuscript. LZ and SY designed the structure of the manuscript. SZ and WZ revised, read, and approved the submitted version. All authors read and approved the manuscript.

## Conflict of Interest

The authors declare that the research was conducted in the absence of any commercial or financial relationships that could be construed as a potential conflict of interest.

## Abbreviations

ALS: amyotrophic lateral sclerosis
AD: Alzheimer disease
ATP: adenosine triphosphate
SV: synaptic vesicle
cGAMP: cyclic GMP-AMP
cGAS: cyclic GMP-AMP synthase
CRL3: cullin3-RING ligase
DAXX: death domain-associated protein
IDR: intrinsically disordered region
PTM: posttranslational modification
LAT: linker for the activation of T cells
LLPS: liquid–liquid phase separation
m^6^A: *N*^6^-methyladenosine
PAS: pre-autophagosomal structure
PML: promyelocytic leukemia
POZ: pox virus and zinc finger
RNP: ribonucleoprotein
SPOP: speckle-type POZ domain protein
CTD: carboxy-terminal domain
TCR: T cell antigen receptor
TF: transcription factor
APC: antigen-presenting cell
PRM: proline-rich motif
ATG: autophagy-related genes
PD-1: programmed cell death protein 1
CTLA-4: cytotoxic T lymphocyte antigen 4
TIM-3: T-cell immunoglobulin and mucin domain 3
TORC1: target of rapamycin complex 1
RBP: RNA-binding protein
DDR: DNA damage response
PARP1: Poly(ADP-ribose) polymerase 1
PARylation: poly(ADP-ribosyl)ation
ssDNA: single-strand DNA
53BP1: p53-binding protein 1
DSB: DNA double-strand breaks
SH3: SRC homology 3
WASP: Wiskott–Aldrich syndrome protein
MATH: N-terminal meprin and TRAF-C homology.
